# Supplementary material for: Folliculin directs the formation of a Rab34–RILP complex to control the nutrient‐dependent dynamic distribution of lysosomes
Source: EMBO Rep. 2016 Apr 13;17(6):823–41. doi: 10.15252/embr.201541382 (PMC4893818; doi:10.15252/embr.201541382)
Supplement: Supplementary file 1 — Expanded View Figures PDF [file EMBR-17-823-s001.pdf]

Expanded View Figures

A

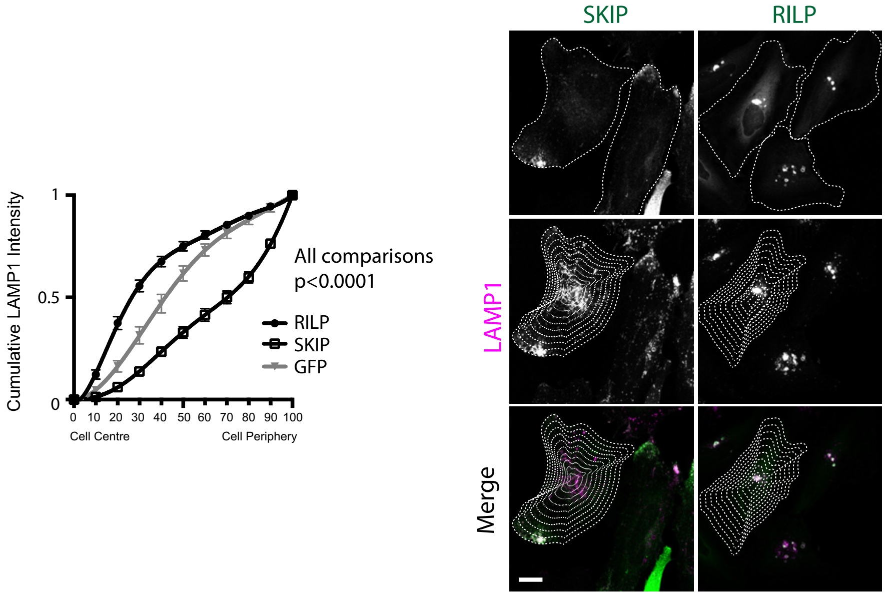

B

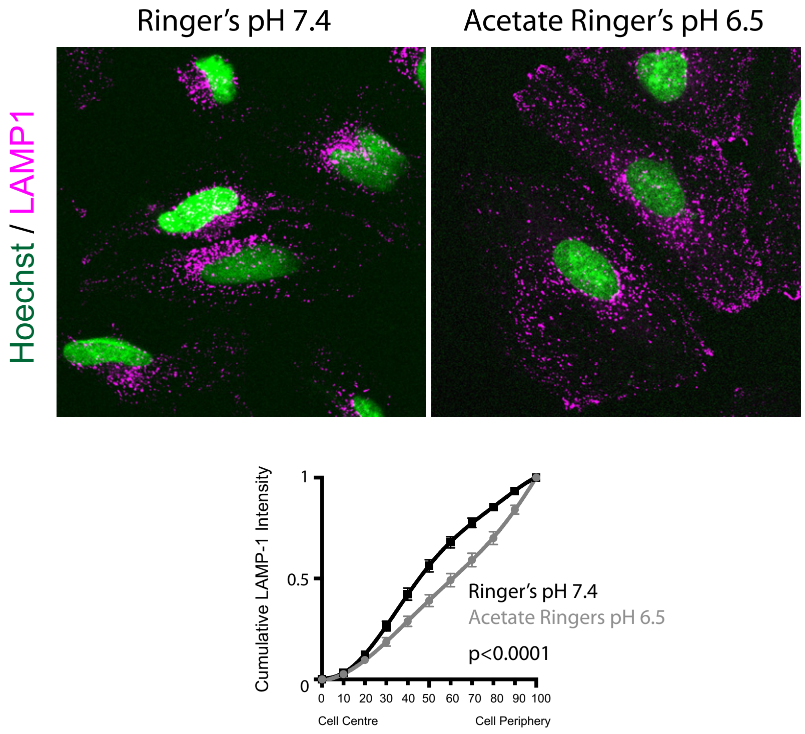

Figure EV1. A cumulative intensity method for quantification of lysosome distribution.

A Graph showing cumulative distribution of LAMP1 intensity (left) in GFP, GFP-RILP or myc-SKIP transfected HeLa cells. Error bars show ± SEM from 20 cells. *P*-value is determined by the extra sum of F-squares test following nonlinear regression and curve fitting. Representative images of GFP-RILP or myc-SKIP transfected HeLa cells stained with LAMP1 (right) illustrating application of cumulative intensity distribution method. Scale bar, 10 μm.

B Immunofluorescence images and graph showing distribution of LAMP1 in HeLa cells treated for 30 min with Ringer's pH 7.4 or Acetate Ringer's pH 6.5. *P*-value is determined by the extra sum of F-squares test following nonlinear regression and curve fitting. Error bars show ± SEM from 3 cells in 3 replicates.

 © 2016 The Authors

Figure EV2. Role of the FNIP proteins in control of lysosome distribution.

A Graphs showing cumulative LAMP1 distribution (top) in cells transfected with non-targeting siRNA or siRNA against FNIP1/2, in normal growth or starvation conditions. Error bars show ± SEM from 30 cells. *P*-value is determined by the extra sum of F-squares test following nonlinear regression and curve fitting. Image of ethidium bromide stained agarose gel (bottom) showing results of RT–PCR experiment with oligos designed to detect FNIP1 and FNIP2 from total RNA preparations using 50 ng of total RNA from cells transfected with siRNA against both proteins.

B Confocal immunofluorescence images showing lysosome distribution and localisation of endogenous FLCN in control cells or cells transfected with siRNA against FNIP1/2.

C Widefield immunofluorescence image of HA-FNIP2 transfected HeLa cell showing co-localisation of endogenous FLCN and HA-FNIP2.

D Western blot of whole-cell lysates showing relative expression of FLCN-HA, HA-FNIP1 and HA-FNIP2 following transfection in HeLa cells. Blot is over-exposed for FLCN-HA to visualise HA-FNIP1/2.

© 2016 The Authors

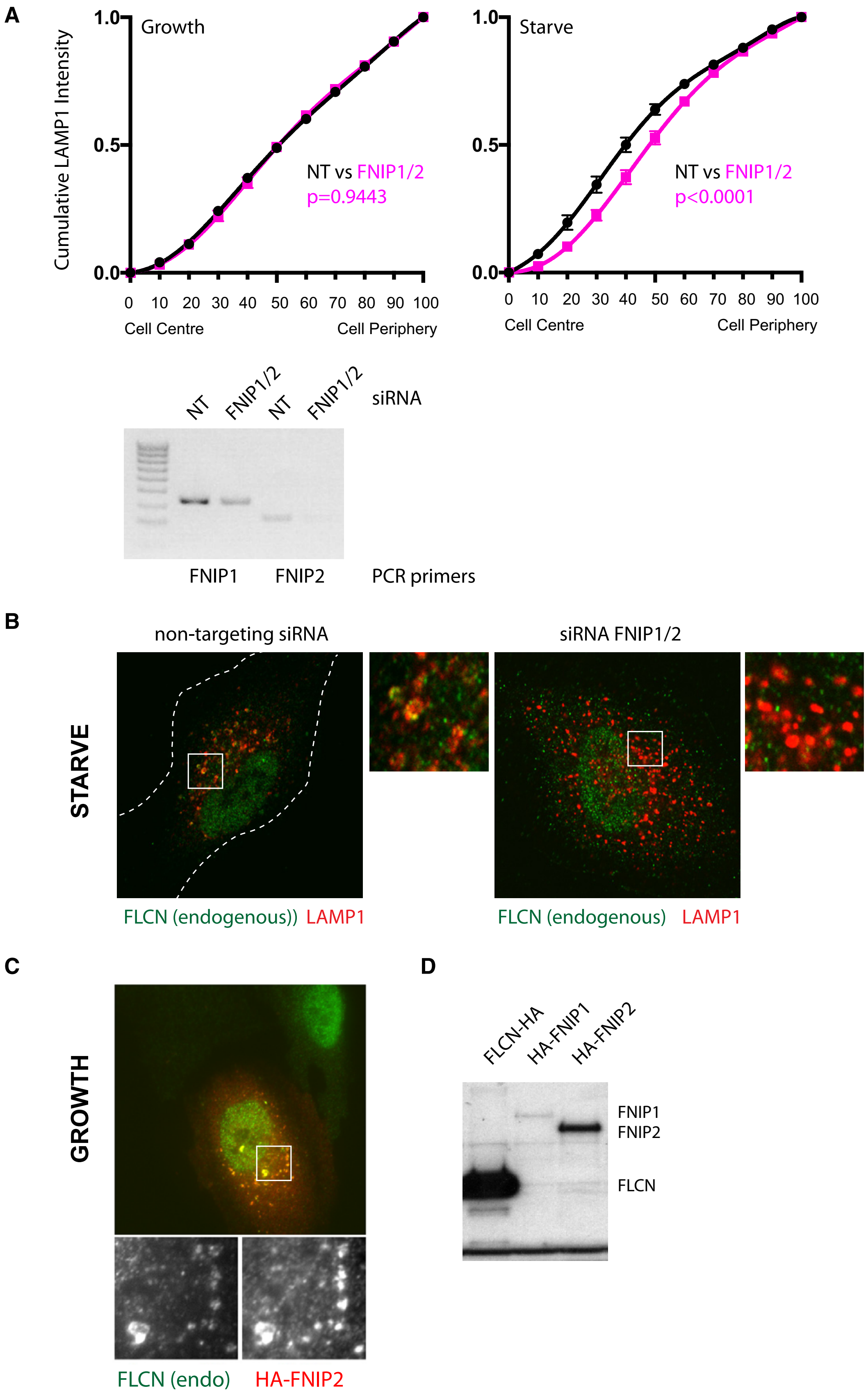

Figure EV2.

 © 2016 The Authors

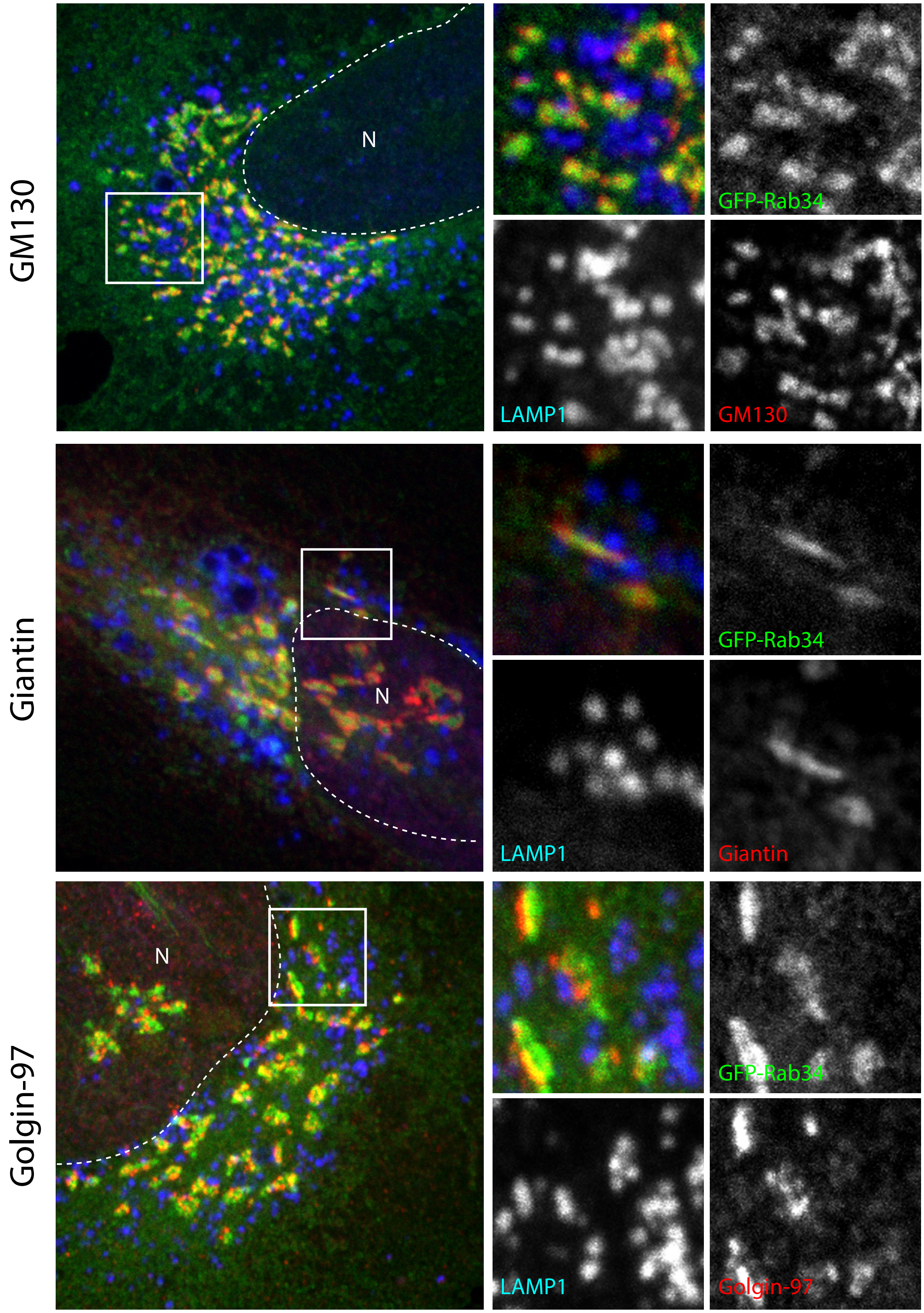

Figure EV3. GFP-Rab34 associates with the Golgi.
Confocal maximum intensity projection immunofluorescence images of Golgi regions of HeLa cells transfected with GFP-Rab34 and co-stained for LAMP1 and GM130 (top), giantin (middle) or golgin-97 (bottom).

© 2016 The Authors

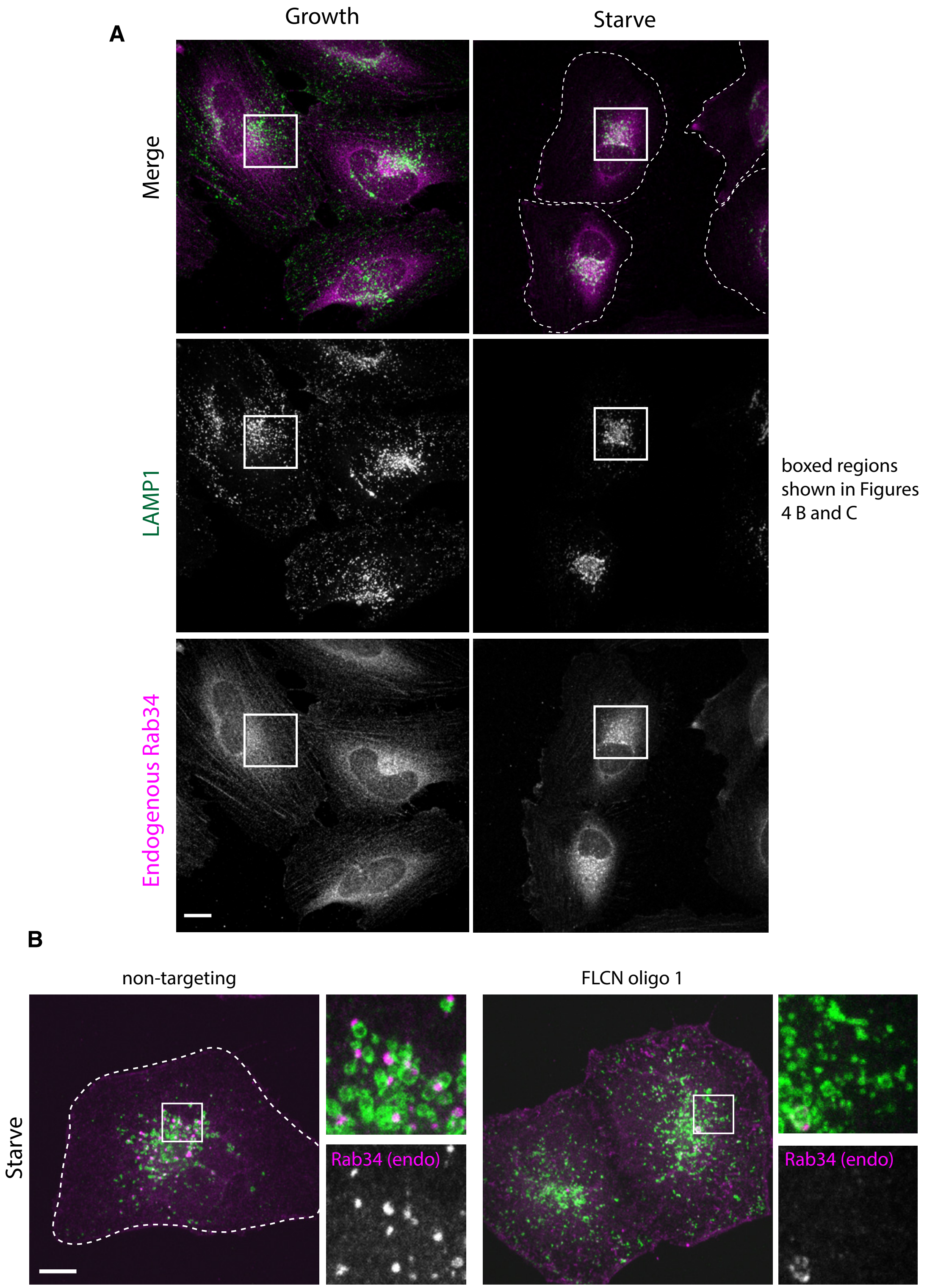

Figure EV4. Localisation of endogenous Rab34.

A Maximum intensity projection images of confocal Z-stacks showing LAMP1 and endogenous Rab34 localisation in HeLa cells under growth and starvation conditions. Boxes highlight regions in zoom panels in Fig 4B. Scale bar, 10 µm.

B Maximum intensity projection images of confocal Z-stacks under starvation conditions showing LAMP1 and endogenous Rab34 localisation in cells transfected with a non-targeting siRNA or when FLCN is depleted. Scale bar, 10 µm.

 © 2016 The Authors

Figure EV5. Targeting of Rab34 and folliculin to mitochondria.

A Confocal immunofluorescence images of Rab34/Rab35-dsRED-Mito transfected HeLa cells showing expected targeting of Rab34-dsRED-Mito and Rab35-dsRED-Mito to mitochondria (labelled with anti-mitochondria antibody). Scale bar, 10 μm.

B Confocal Immunofluorescence images of HeLa cells transfected with Rab35-dsRED-Mito FLCN-GFP and HA-FNIP2. White arrows highlight FLCN-GFP/HA-FNIP2 co-localisation.

C Confocal immunofluorescence images of Rab34-dsRED-Mito (WT or Q111L) and FLCN-GFP (without HA-FNIP2) transfected HeLa cells showing recruitment to mitochondria. Scale bar, 10 μm.

D SIM super-resolution image showing a single plane of a region of a Rab34 (Q111L)-dsRED-Mito/FLCN-GFP/HA-FNIP-2 transfected cell stained with LAMP1 antibodies. Scale bar, 2 μm.

© 2016 The Authors

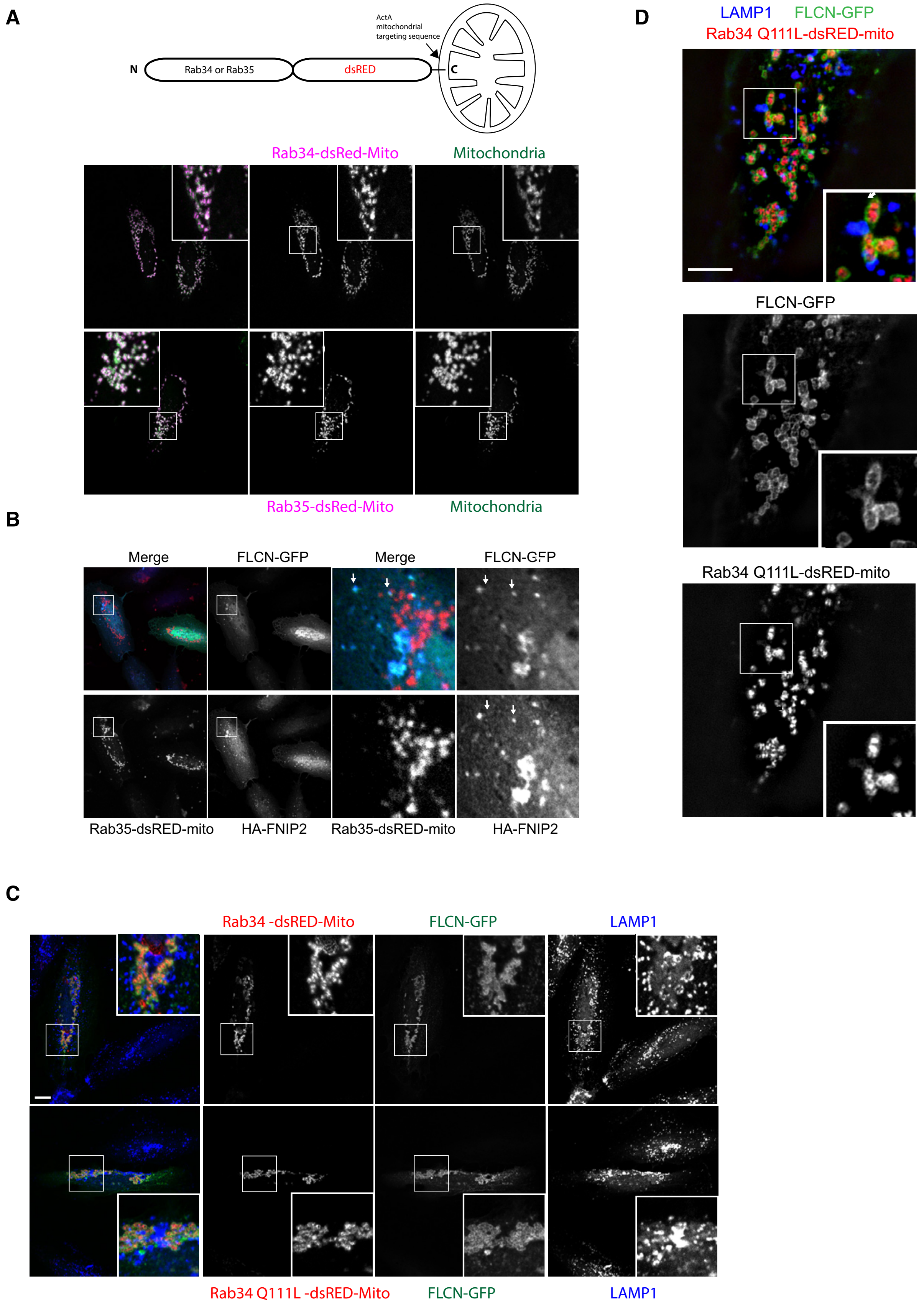

Figure EV5.

 © 2016 The Authors

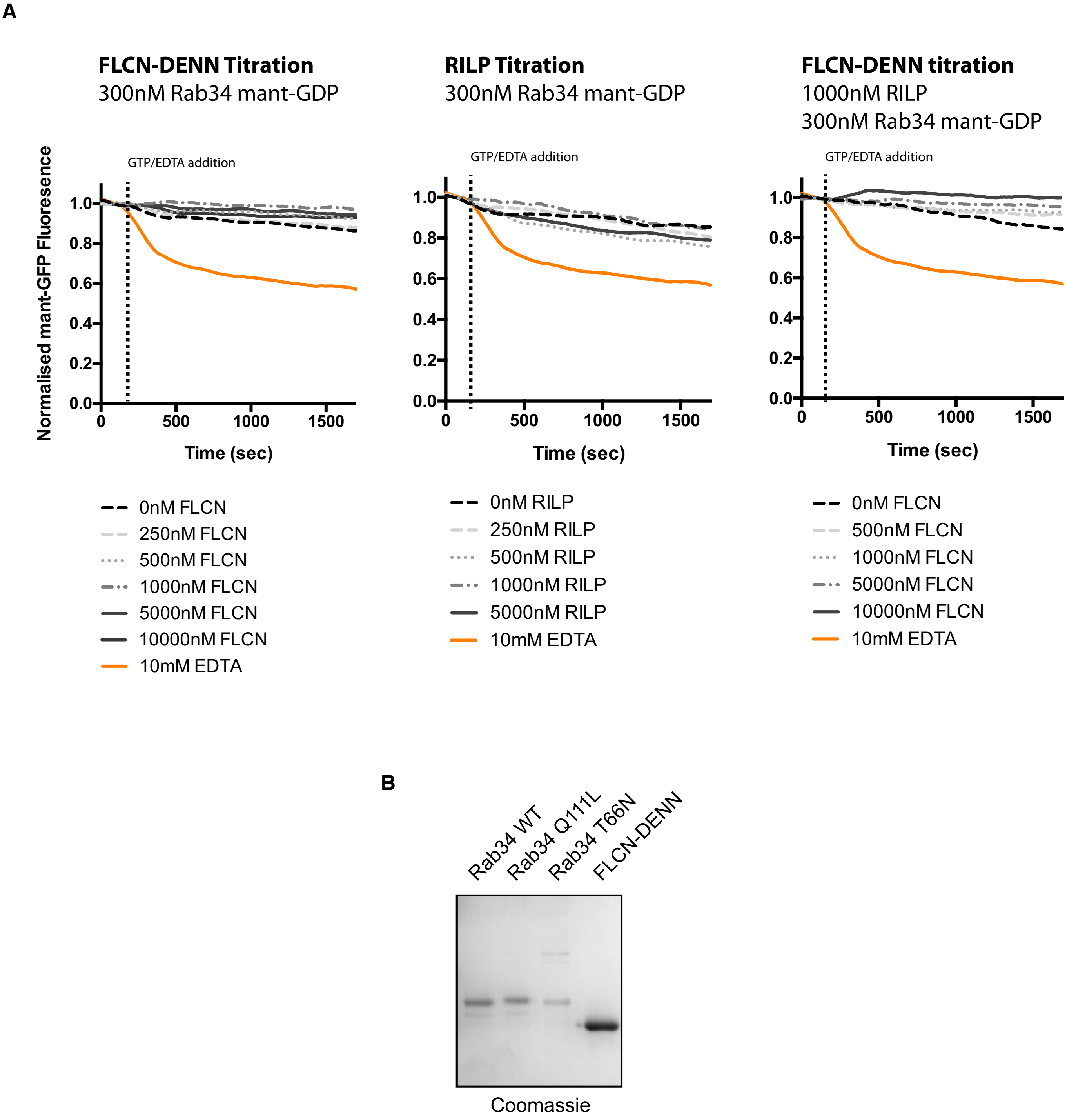

Figure EV6. ***In vitro*, the FLCN DENN domain does not possess Rab34 GEF activity.**

A Graphs showing results of Rab34 GEF assays; 300 nM Rab34 loaded with mant-GDP and incubated with various combinations and concentrations of FLCN-DENN and RILP. GTP was added at a concentration of 0.3 mM or EDTA at a concentration of 10 mM at the 2 min time point. Data were acquired at 10-s intervals for 25 min. Curves are mean of duplicate samples and are smoothened using a 6-point rolling average to reduce instrument noise. The same EDTA curve is reproduced across all three graphs for comparison.

B Coomassie stained SDS–PAGE gel showing samples of His-tagged Rab34 WT, Q111L, T66N and FLCN-DENN domain proteins used in this study.

© 2016 The Authors

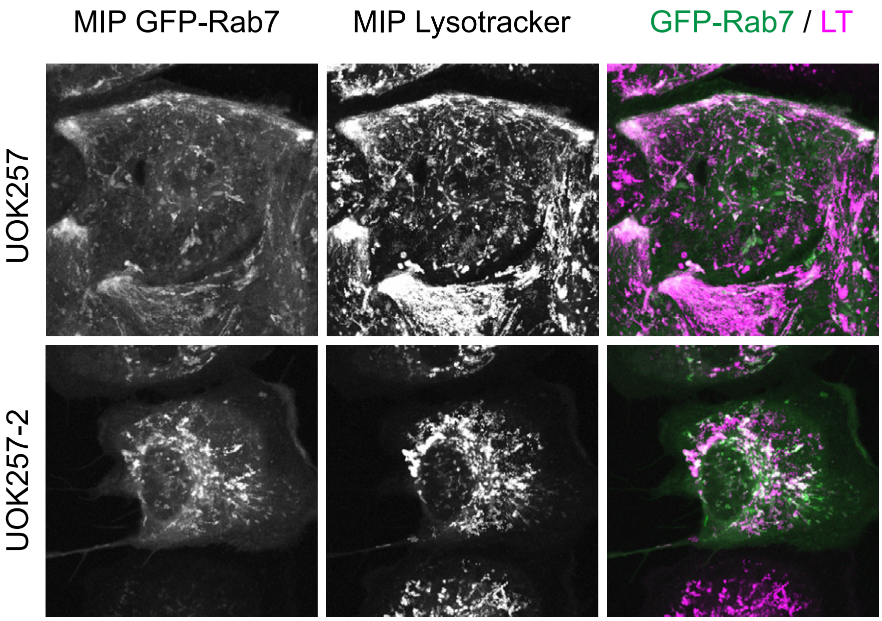

Figure EV7. Lysosome dynamics in UOK257 and UOK257-2 cells.
Maximum intensity projection images from 120 frames of Movies EV5 and EV6 highlighting GFP-Rab7 (green) and Lysotracker-Red (magenta) dynamics in UOK257 and UOK257-2 cells during the course of the movie.

© 2016 The Authors
